# An assessment of orofacial clefts in Tanzania

**DOI:** 10.1186/1472-6831-11-5

**Published:** 2011-02-02

**Authors:** Mange Manyama, Campbell Rolian, Japhet Gilyoma, Cassian C Magori, Kilalo Mjema, Erick Mazyala, Emmanuel Kimwaga, Benedikt Hallgrimsson

**Affiliations:** 1 Department of Anatomy, Bugando University College of Health Sciences, Mwanza, Tanzania; 2 Department of Anatomy and Cell biology, University of Calgary, Calgary, Canada; 3 Department of Surgery, Bugando Medical Centre, Mwanza, Tanzania

## Abstract

**Background:**

Clefts of the lip (CL), the palate (CP), or both (CLP) are the most common orofacial congenital malformations found among live births, accounting for 65% of all head and neck anomalies. The frequency and pattern of orofacial clefts in different parts of the world and among different human groups varies widely. Generally, populations of Asian or Native American origin have the highest prevalence, while Caucasian populations show intermediate prevalence and African populations the lowest. To date, little is known regarding the epidemiology and pattern of orofacial clefts in Tanzania.

**Methods:**

A retrospective descriptive study was conducted at Bugando Medical Centre to identify all children with orofacial clefts that attended or were treated during a period of five years. Cleft lip and/or palate records were obtained from patient files in the Hospital's Departments of Surgery, Paediatrics and medical records. Age at presentation, sex, region of origin, type and laterality of the cleft were recorded. In addition, presence of associated congenital anomalies or syndromes was recorded.

**Results:**

A total of 240 orofacial cleft cases were seen during this period. Isolated cleft lip was the most common cleft type followed closely by cleft lip and palate (CLP). This is a departure from the pattern of clefting reported for Caucasian and Asian populations, where CLP or isolated cleft palate is the most common type. The distribution of clefts by side showed a statistically significant preponderance of the left side (43.7%) (χ^2 ^= 92.4, p < 0.001), followed by the right (28.8%) and bilateral sides (18.3%). Patients with isolated cleft palate presented at very early age (mean age 1.00 years, SE 0.56). Associated congenital anomalies were observed in 2.8% of all patients with orofacial clefts, and included neural tube defects, Talipes and persistent ductus arteriosus.

**Conclusions:**

Unilateral orofacial clefts were significantly more common than bilateral clefts; with the left side being the most common affected side. Most of the other findings did not show marked differences with orofacial cleft distributions in other African populations.

## Background

Clefting of the lip (CL), the palate (CP), or both (CLP) are the most common orofacial congenital malformations found among live births, accounting for 65% of all head and neck anomalies [1]. Aetiological factors for the majority of cleft lips and/or cleft palates include genetic and environmental factors, their interaction effects, as well as phenotypic variability that occur during early development [2-5]. It is estimated that orofacial clefts occur in ~1/700 to ~1/1000 live births in different populations around the world, with substantial variability related to geographic origin, ethnicity, and socioeconomic conditions (Table 1) [6,7]. About 70% of orofacial cleft cases are nonsyndromic, i.e. with affected individuals showing no other physical or developmental anomalies [8]. The frequency and distribution of orofacial clefts also varies widely among different populations (Table 1). Although some of these variations can be attributed to differences in the classification of orofacial clefts, populations of Asian or Native American origin tend to show the highest prevalence, with Caucasian populations showing intermediate and African populations the lowest prevalence [7,9,10]. Most studies of African populations have reported isolated cleft lip as the most common cleft type, and cleft palate the least (e.g. [11,12]), which may represent a departure from the pattern of clefting reported for non-African populations, where CLP and CP are most common (Table 1).

**Table 1:** Incidence and distribution of orofacial clefts among different populations

Population	Prevalence	Frequency (%)			Frequency of associated congenital anomalies
		CL	CP	CLP	
Europe					
Scotland [29]	1.53	17.8	51.4	30.8	32.9
Sweden [16]	1.70	26.5	38.8	34.7	21.0
EUROCAT [8,30,31]	1.52	22.1	39.5	38.4	36.5
Asia					
Japan [32]	1.44	32.1	24.8	43.2	21.1
China [33]	1.66	31.4	15.4	53.2	14.4
Pakistan [34]	1.91	41.9	23.9	34.2	17.9
Africa					
Zambia [11]	0.24	58.3	3.9	37.8	n/a
Nigeria [24]	0.37	49.2	18.9	31.9	18
Kenya [14]	n/a	30.6	8.5	60.9	8.2

Orofacial clefts have long been known to be associated with other congenital defects, though the frequency and type of associated malformations observed varies considerably across studies [13]. Observed differences in frequency may be attributed in part to the methods of data collection, with studies based on the review of birth certificates and hospital records reporting lower incidences than birth registry based studies, or those that record patients referred to their institutions for treatment. For example, a recent study by Wanjeri and Wachira [14], based on a retrospective review of hospital records in Kenya, revealed a frequency of 8.2% associated congenital anomalies in patients with orofacial clefts. In contrast, Calzolari et al [8] found that 29.2% of CL/P cases were associated with other congenital deformities based on a survey of 23 European birth registries, while a substantially higher incidence of 63.4% associated anomalies was reported by Shprintzen et al [15] based on a review of 1000 patients who visited the Center for Craniofacial Disorders at the Montefiore Medical Center in New York. Some studies have found that associated malformations are more frequent in infants with CLP than in infants with CL only [8], and several of these associated anomalies may require follow-up or further treatment [16]. Defects in the musculoskeletal, cardiovascular, and central nervous systems are most frequently associated with orofacial clefts [17]. The co-existence of CL/P with other congenital anomalies highlights the importance for clinicians to screen for associated congenital anomalies in patients with CL/P as the potential functional outcomes may be affected during treatment and rehabilitation (e.g. [16]).

Although the epidemiology of cleft lip and palate has been studied extensively in different parts of the world (Table 1) [18,19], there is little and often conflicting information on the epidemiology of cleft deformities in African populations. Some reports from African studies have suggested that the pattern of orofacial clefts differs significantly from that reported in other populations [11,20,21]. To date, little information has been available regarding the epidemiology of orofacial clefts in Tanzania. The main objective of this study is to establish the frequency, laterality, sex and geographical distribution of orofacial clefts and their associated congenital anomalies among patients attending Bugando Medical Centre in Mwanza, Tanzania, from 2004 to 2009. The distribution of various types of orofacial clefts, differences between sexes, and the frequency of associated anomalies and syndromes were evaluated with the aim of understanding the clinical pattern of these patients in Tanzania, and contributing to the global literature on the epidemiology of orofacial clefts.

## Methods

A retrospective descriptive study was conducted at Bugando Medical Centre, located in the northwest region of Mwanza, Tanzania, to identify all children with orofacial clefts that attended or were treated from October 2004 to July 2009. Bugando Medical Centre (BMC) is one of four referral hospitals in the country, serving a population of approximately 10 million people in five regional districts. Most patients with orofacial clefts in the surrounding regions are usually referred to this hospital as it is the only centre that offers surgical expertise to repair orofacial clefts on the North-Western part of Tanzania. Cleft lip and/or palate records were obtained from patient files in the Hospital's Departments of Surgery, Paediatrics and Medical Records. Patient file notes are usually written by medical officers and surgeons from the time of hospital admission to discharge. Age at presentation, sex, region of origin, type and laterality of the cleft were recorded. Additionally, presence of associated congenital anomalies or syndromes was recorded. Orofacial cleft cases that lacked some of the above information (e.g. type of cleft, laterality) were excluded. Most of the associated congenital anomalies reported were of a type that can be readily observed by medical officers and/or surgeons, as genetic services are very limited at BMC. The study was reviewed and approved by the institutional review boards of both Bugando University College and the University of Calgary, in accordance with the dual affiliation of the first author.

Data were recorded and entered into SPSS version 11.5. Cleft type was classified as follows: 1. CL: Cleft lip and alveolus (right, left, bilateral), 2. CLP: Cleft lip and palate (complete; right, left, bilateral), 3. Cleft palate (soft, hard, and submucous). Frequencies were obtained for different types of clefts in relation to the total number of orofacial clefts. Deviations from expected frequencies (e.g. by laterality or sex) were analysed using contingency tables and Chi-square analyses.

## Results

A total of 240 cases with orofacial cleft were seen between October 2004 and July 2009. Among these, 14 cases were admitted in the Paediatrics Department with other medical conditions; orofacial cleft not being the primary reason for hospital admission. About 60 additional cases with orofacial clefts seen at BMC during the study period had incomplete patient files, and thus were not included in the study. The majority of cases came from Mwanza region, followed by Mara and Shinyanga regions (Table 2). Some cases from Kigoma and Kagera were refugees from Burundi and the Democratic Republic of Congo.

**Table 2:** Distribution of orofacial clefts according to type, sex, laterality, region of origin and deviations from expected frequencies

Parameter	n (%)	Type of Cleft			
		CL (%)	CP (%)	CLP (%)	X^2 ^(p)
**Clefts cases**	240(100)	240(100)	28(11.7)	94(39.1)	
**Sex**					
Male	127(52.9)	56(47.5)	16(57.1)	55(58.5)	
Female	113(47.1)	62(52.5)	12(42.9)	39(41.5)	2.76(0.247)
**Laterality**					
Right	69(28.8)	37(31.5)	6(20.7)	26(27.9)	
Left	105(43.7)	62(52.5)	6(20.7)	37(39.8)	
Midline	22(9.2)	2(1.6)	16(55.2)	4(4.4)	
Bilateral	44(18.3)	17(14.4)	1(3.4)	26(27.9)	92.4(p < 0.001)
**Region of origin**					
Mwanza	127(52.9)				
Mara	42(17.5)				
Shinyanga	25(10.4)				
Kagera	17(7.1)				
Kigoma	13(5.4)				
Tabora	11(4.6)				
Singida	5(2.1)				

The most common cleft type was isolated cleft lip (CL), constituting 49.2% of all cleft deformities (Table 2). Clefts of both lip and palate (CLP) and isolated cleft palate (CP) constituted 39.2% and 11.7% of cleft deformities respectively. This pattern is broadly similar to other series in African countries, and differs significantly from the distribution of orofacial clefts in low and middle income countries in other parts of the world (Table 3). Overall, males were slightly more affected than females among all clefts with a frequency of 52.9% and 47.1% respectively. Combined cleft lip and palate (CLP) showed the highest frequency (58.5%) in males while isolated cleft lip (CL) was higher in females (52.5%). However, the association between cleft type and sex was not statistically significant (χ^2 ^= 2.79, df = 2, p = 0.247). In contrast, the association between cleft type and laterality was statistically significant (χ^2 ^= 92.4, df = 6, p < 0.001). The distribution of clefts by side showed a preponderance of the left side (43.7%), followed by right side (28.8%) and bilateral cases (18.3%). Midline clefts accounted for only 10% of all cleft cases. About 2.8% of cleft cases were associated with congenital anomalies, including two cases each of Hydrocephalus, *Talipes equinovarus*, *Spina bifida*, and one case of Persistent *Ductus arteriosus*.

**Table 3:** Distribution of clefts by type in small-scale, hospital/clinic-based epidemiological studies in African and other low- and middle income countries.

	Total Cases	CL(%)	CP(%)	CLP(%)	X^2^	P
**Africa**						
Tanzania (this study)	240	49.2	11.7	39.2	1.92	-
Kenya [4]	68	51.5	5.9	42.6	6.97	0.03
Kenya [12]	368	52.7	5.7	41.6	1.24	ns
Uganda [27]	47	40.4	12.8	46.8	2.47	ns
Nigeria [35]	93	39.8	15.1	45.2	6.85	0.03
Nigeria [24]	360	49.2	18.9	31.9	13.81	< 0.005
Zambia [11]	331	58.3	3.9	37.8	1.74	ns
Zimbabwe [36]	57	42.1	17.5	40.4		
						
**Asia**						
China [37]	637	26.2	15.1	58.7	42.21	< 0.001
Iran [38]	119	29.4	25.2	45.4	17.06	< 0.001
Pakistan [34]	117	41.9	23.9	34.2	8.96	0.01
Thailand [39]	153	23.5	20.9	55.6	26.41	< 0.001
						
**South America**						
Brazil [40]	154	33.1	14.3	52.6	9.95	< 0.01

A wide range of age at presentation was observed in this study (range 1 month to 18 years), with about 33% of all cleft cases presenting at over one year of age. Patients with isolated cleft palate presented earlier (mean age 1.00 years, SE 0.56) as compared to those with isolated cleft lip(mean age 2.39 years, SE 0.27 ) or cleft lip and palate (mean age 1.96 years, SE 0.30); however, these differences were not statistically significant (Kruskal-Wallis ANOVA K = 3.28, p = 0.19).

## Discussion

### Study Limitations

The aim of this retrospective study was to report on key aspects of the epidemiology of orofacial clefts in Tanzania, based on a review of hospital records at Bugando Medical Centre in Mwanza. Retrospective studies are usually small, based on clinic records, subject to underreporting, and may suffer from multiple sources of ascertainment bias (e.g. socioeconomic factors). While prospective studies are ideal, they are not currently feasible in Tanzania. Even so, ongoing studies of the genetics of CLP in East-Africa require analysis and interpretation of the existing clinical records to determine what they can reveal about the epidemiology of CLP in this region.

In this study, potential sources of underreporting at BMC include the distance and/or cost of travel to the hospital and referrals to other treatment centres closer to the patient's area of residence. For instance, although patients in this study came from the seven regions served by BMC as a referral hospital, the majority came from the regions closest to the hospital, namely, Mwanza, Mara and Shinyanga. Therefore, as was recently observed in another study in Kenya [22], distance and/or accessibility to the hospital may play a significant role in determining the number of patients seen per region, or whether patients from particular regions are seen at all. A number of patients with orofacial clefts from Kagera and Kigoma were refugees, or born to refugees, from Burundi and the Democratic Republic of Congo. Kagera and Kigoma are regions that border Burundi and the DRC, respectively, and have been receiving refugees due to political instability in these neighbouring countries.

Bugando Medical Centre provides surgical treatment and other supportive care like speech therapy to patients with orofacial clefts. Due to differential reporting among surgeons and the fact that some of the records were missing key post operative information, we did not investigate the fate or outcome (e.g. morbidity, complications, mortality etc) of these patients after surgery.

### Demographics and Epidemiology of Orofacial Clefts in Tanzania

At BMC, isolated cleft lip was the most common cleft type, followed closely by clefts of both the lip and palate. Similar findings have been observed in other African studies (Tables 1 and 3). For example, Spritz *et al *[12] found that the proportion of CL was higher in the Rift Valley region of Kenya than in other places in Africa, partly due to the preponderance of an atypical cleft lip variant that was not specifically identified in the patient records at BMC. In contrast, most studies in Caucasian and Asian populations have reported higher frequency of isolated cleft palate and cleft of both palate and lip (Table 1) [8,16,23]. Although some of this variation is attributable to differences in study design, our analysis of comparable, small, retrospective or hospital-based studies in low and middle income countries in Africa, Asia and South America suggests this variation may reflect a biological phenomenon (Table 3). In other words, not only the prevalence but also the distribution of cleft types may be racially and ethnically determined [22]. Alternatively, the low number of patients with isolated cleft palate in this and other African studies may reflect a higher mortality rate in this group associated with functional difficulties during feeding in young infants [24,25].

The sex distribution of isolated cleft lips showed a skew towards females (Table 2), which contrasts with results of similar clinic-based studies in Africa [20,22,24]. The observed pattern of male predominance in clefts of both lip and palate are in accordance with literature data on Caucasian populations [26]. In contrast, isolated cleft palate was more common in males, which agrees in part with findings from Kenya [22]but differs from most Caucasian series in which clefts of the palate were found to occur more frequently in females [9,26].

The age at presentation in our study does not support the hypothesis that patients with orofacial clefts in low and middle income countries tend to present at later age due to unavailability of specialized medical facilities [20]. As has been documented in Uganda [27], a neighbouring country with a similar ethnic and socio-economic profile, we expected that a higher proportion of patients would present at an older age, as current levels of medical care in Tanzania are not able to meet the demand for orofacial cleft treatment. However, our results are more similar to another clinic-based study in Kenya, in which a greater proportion of patients with clefts presented under the age of 1 year [22]. A potential explanation for the observed age distribution could be improvements in the Tanzanian healthcare and health education systems in recent years, as well as the occasional availability of programs that support treatment of orofacial clefts supported by AMREF and mining companies in this region. Among different orofacial cleft types, patients with isolated cleft palate presented earlier than those with isolated cleft lip. This could be explained by the fact that young patients with isolated cleft palate and those with cleft lip/palate often do have trouble feeding; hence their parents are likely to bring the children to hospital earlier. Similar patterns have also been found elsewhere [24,25].

The result from this study shows the frequency of patients with orofacial clefts and associated congenital anomalies to be 2.8%. Other studies have reported that this frequency can range from as low as 4.3% [28] to as high as 63.4% [15]. The wide range of reported frequencies of associated congenital anomalies have been attributed in part to the methods of data collection, with lower incidence being reported by studies that have reviewed birth certificates (not birth registries) than studies that account for patients referred to their institutions for treatment [16]. The observed frequencies of associated congenital anomalies in this study probably reflect differential mortality rates among cleft cases associated with anomalies in vital internal organs. The few anomalies observed included neural tube defects, clubfoot and persistent ductus arteriosus, consistent with previous studies [16,17].

## Conclusions

In this preliminary study, we found that unilateral clefts were more common than bilateral clefts. Unilateral clefts showed preponderance for the left side. Most of the other findings from our study regarding distribution of orofacial clefts were similar to other African populations. Owing to the nature of this hospital-based retrospective study, it was not possible to estimate the true prevalence of orofacial clefts and their associated congenital anomalies in Tanzania at this time. Further large population- and birth registry-based studies are needed to obtain more representative results regarding both the prevalence and frequency of associated anomalies for orofacial clefts in Tanzania, and more broadly in Africa.

## Competing interests

The authors declare that they have no competing interests.

## Authors' contributions

MM made substantial contributions to the conception and design of this study, participated in data collection, statistical analysis and interpretation of results, drafted and revised the final manuscript, and read and approved the final manuscript. CR contributed substantially to statistical analysis and draft of the manuscript. JG and CM participated in the design and data analysis. KM, EM and EK made substantial contribution to the data collection and analysis. BH made substantial contribution to the statistical analysis and draft of the manuscript. All authors have read and approved the final manuscript.

## Pre-publication history

The pre-publication history for this paper can be accessed here:

http://www.biomedcentral.com/1472-6831/11/5/prepub
